# Global research trends and hotspots of PI3K/Akt signaling pathway in the field of osteoarthritis: A bibliometric study

**DOI:** 10.1097/MD.0000000000033489

**Published:** 2023-04-14

**Authors:** Rui Zhang, Xiaoqin Deng, Quan Liu, Xintian Zhang, Xinxin Bai, Shaohuang Weng, Min Chen

**Affiliations:** a Department of Orthopedic, Fujian Medical University Union Hospital, Fuzhou, China; b Department of Pharmaceutical Analysis, School of Pharmacy, Fujian Medical University, Fuzhou, China.

**Keywords:** bibliometric study, osteoarthritis, PI3K/Akt, signaling pathway

## Abstract

The phosphatidylinositol 3-kinase/protein kinase B (PI3K/Akt) signaling pathway has gradually become a new target for the treatment of osteoarthritis (OA). Numerous studies of PI3K/Akt signaling in OA have been published in the past few years. By analyzing these research characteristics and qualities, we aimed to reveal the current research focus and emerging trends in PI3K/Akt signaling in OA. We searched the Web of Science database for relevant articles concerning the PI3K/Akt signaling pathway in OA published from inception to October 31, 2022. The following data were extracted: author name, article title, keywords, topic, publication country/region, institution, publication journal, journal impact factor, number of times cited, and H-index. VOSviewer and Excel 2019 were used to conduct the bibliometric study and visualize the analysis. A total of 374 publications were included in this study. In all selected articles, “orthopedics” was the dominant topic (252 of 374, 67.38%). The most productive year was 2021. *Frontiers in Pharmacology* published the most articles. The People’s Republic of China has published the most articles worldwide. The top 5 keywords were “OA,” “expression,” “apoptosis,” “chondrocytes,” and “inflammation.” The keywords “autophagy,” “mitochondrial dysfunction,” “inflammatory response,” “cartilage degeneration,” and “network pharmacology” have increased in recent years. Our study showed a growing trend in published articles related to the PI3K/Akt signaling pathway in OA. Inflammatory response, cartilage degeneration, and apoptosis remain central topics in the field. Research on autophagy, mitochondrial dysfunction, and network pharmacology is on the rise, and the focus on PI3K/Akt will continue to increase.

## 1. Introduction

Osteoarthritis (OA) is a chronic inflammatory disease caused by the combined effect of various pathogenic factors. It is mainly characterized by cartilage degeneration and destruction, and the main symptoms are local joint swelling and pain accompanied by bouncing, effusion, functional impairment, and limited activity.^[1]^ OA tends to occur in joints with frequent friction, a heavy negative load, and easy damage, with a prevalence that is higher in middle-aged and elderly people as well as obese groups. With societal development and the accelerated pace of life, the disease has gradually affected younger individuals and has a more obvious significant growth trend, with serious implications for individuals and society.^[2]^ Physical, drug, and surgical therapies are often used in the clinical treatment of patients with OA based on the principles of pain relief and disease control. However, each has certain defects and great limitations, and patients tend to have a poor prognosis with a high risk of relapse. Effective radical treatment measures are lacking.^[3]^ Therefore, the pathophysiology and regulatory mechanisms of OA require further exploration.

Phosphatidylinositol3-kinase (PI3K) is a signaling protein with a catalytic activity that is widely present in various cells of the body. It can act on downstream effector molecules and specifically catalyze PI3K. It is involved in cell proliferation, migration, apoptosis, and other life activities as an extracellular messenger involved in inflammatory, allergic, and other reactions, and is the initiation factor of the PI3K/protein kinase B (Akt) pathway.^[4]^ Akt, a target protein with a molecular weight of approximately 57 kDa, is divided into 3 subtypes with highly consistent activation and sequence homology: PKB-α, PKB-β, and PKB-γ. There is a pleckstrin homology domain, a catalytic domain in the middle, and a C-terminal domain that is composed of the regulatory domain of PI3K and is the main effector molecule downstream of PI3K. Phosphatidylinositol 3-kinase/protein kinase B (PI3K/Akt) is an important autophagy signaling pathway in cells. Activation of this pathway can regulate cell growth and metabolism, inhibit the activity of various autophagy-related proteins, affect the activity of downstream effector molecules, and reduce the autophagic ability of cells. It plays a key role in inhibiting cell apoptosis and is closely related to cell growth and metabolism as well as the occurrence and development of various diseases.^[5]^

OA is a complex disease involving multiple molecular mechanisms that affect the whole joint with synovial inflammation, cartilage degeneration, and subchondral bone sclerosis. Currently, PI3K/AKT signaling-based intervention strategies for OA can be divided into 2 main categories: inhibition of PI3K/AKT signaling attenuates joint damage due to OA by restoring cartilage homeostasis, enhancing autophagy, and suppressing inflammatory responses.^[2]^ Activation of PI3K/AKT signaling may play an anti-arthritic role by promoting chondrocyte proliferation and reducing apoptosis. Thus, this pathway has gradually become a new target for the treatment of OA.^[6,7]^ The mechanism of pathway activation in OA should be studied to explore new methods for preventing and treating OA.

According to the literature, a bibliometric analysis uses statistical analysis to analyze the distribution structure, quantitative relationship, and change in relevant information quantitatively and qualitatively in the literature to guide future research directions.^[8]^ VOSviewer is a Java-based scientific bibliometric knowledge network analysis software. Through clustering, superposition, density, and other unique perspectives, a visual analysis of relevant literature was performed to show the research status, hot spots, and trends in a certain field from multiple perspectives.^[9]^ To date, bibliometric analyses have been widely applied in multiple fields such as cancer,^[10]^ nursing,^[11]^ OA,^[12]^ and signaling pathways.^[13]^ In recent years, numerous studies have focused on the PI3K/Akt signaling pathway in OA. However, to the best of our knowledge, there have been no relevant bibliometric studies.

Here we conducted a bibliometric analysis of relevant articles concerning the PI3K/Akt signaling pathway in OA. While analyzing the characteristics and qualities of these studies, we aimed to elucidate the growing trends and future direction of the PI3K/Akt signaling pathway in OA.

## 2. Materials and methods

### 2.1. Data source and search strategy

In this bibliometric study, we searched the Web of Science (WOS) database (https://www.webofscience.com) for relevant studies. The databases included the Social Sciences Citation Index, Arts & Humanities Citation Index, Conference Proceedings Citation Index-Science, Conference Proceedings Citation Index-Social Science & Humanities, and Emerging Sources Citation Index. The WOS database is an internationally recognized database that reflects the level of scientific research. It currently contains >8800 international and high-impact academic journals in the natural sciences and covers 176 fields of natural science, engineering, and biotechnology, including chemistry and chemical engineering, materials science, engineering, computer science, physics, environmental science and engineering, food science and technology, genetics, genetics, zoology, botany, and microbiology. The retrieval period was from its inception to October 31, 2022. The search strategy used was the following medical subject headings keywords: “PI3K/Akt,” “PI3K/Akt signaling,” “PI3K/Akt pathway,” and “osteoarthritis.”

### 2.2. Article selection

**Figure 1.**
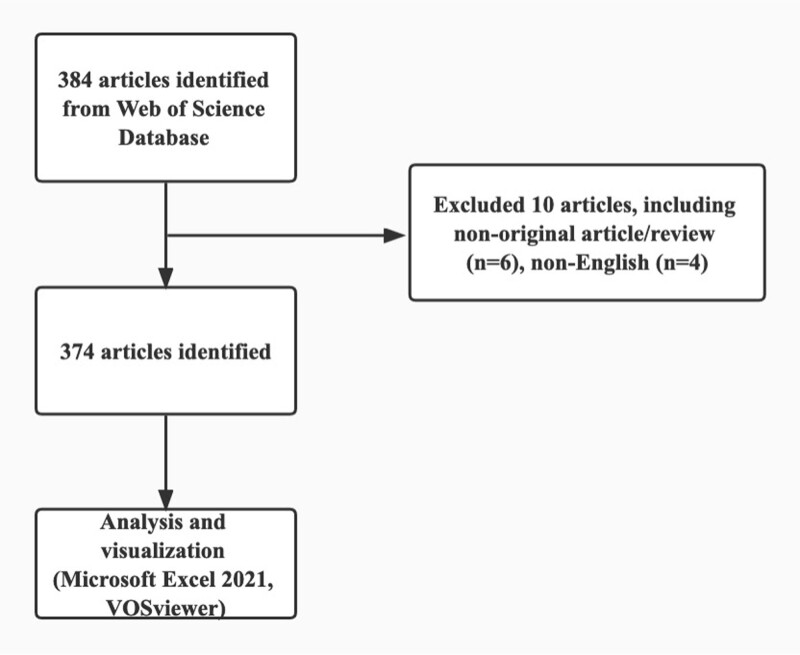
Flow diagram of the study.

Two independent reviewers (X.D. and Q.L.) screened the titles, abstracts, and full texts of the retrieved studies. Discrepancies were adjudicated by a third reviewer (X.Z.). Only original articles and reviews published in English were included (Fig. 1).

### 2.3. Data collection

After relevant articles were selected, the following data were extracted for bibliometric analysis: author name, article title, keywords, topic, publication country/region, institution, publication journal, the journal impact factor (IF) in the publication year, total times cited, and H-index. The IF, one of the data points in the Journal Citation Reports produced by Thomson Reuters, is calculated as follows: total number of citations of articles published in the previous 2 years divided by the total number of articles published in the same journal in the Journal Citation Reports year. The IF is an internationally accepted journal evaluation index. The H-index, also known as the H-factor, is a new method for evaluating academic achievement. A researcher’s H-index is when he/she has at most H papers that are referred to at least H times. The H-index can accurately reflect an individual’s academic achievement. The higher the researcher’s H-score, the greater the impact of his or her papers.

### 2.4. Statistical analysis

The software used in this study included Microsoft Excel 2021 (Microsoft Corporation, USA) and VOSviewer 1.6.16 (Leiden University, Leiden, Netherlands). Microsoft Excel 2021 was used to collect and analyze the number of publications, journals, publication year, publication country/region, and topic. The downloaded data were imported into VOSviewer for further analysis. The co-citation of references and authors and the co-occurrence of keywords were visualized.

## 3. Results

### 3.1. Overview of included publications

According to our inclusion and exclusion criteria, a total of 374 publications were retrieved from WOS (Supplemental Digital Content, http://links.lww.com/MD/I774). The total number of citations was 7018 (6300 without self-citations). The average number of citations for the included articles was 18.76. The H-indices of the enrolled publications related to “PI3K-Akt” and “osteoarthritis” were 42.

### 3.2. Top articles

The top 10 most-cited articles are listed in Table 1. Of them, 8 were original articles and 2 were review articles. They were all published after 2010, with a mean citation number of 122.1 and a mean citation density of 19.715. Only 2 reviews were published in 2020, but they obtained a larger citation density in the top 10 most-cited articles (42.27% and 38.67%, respectively).

**Table 1:** Top 10 most-cited articles in this study.

Rank	Authors	Title	Publication year	Citations	Citation density
1	Xue et al	Inhibition of PI3K/AKT/mTOR signaling pathway promotes autophagy of articular chondrocytes and attenuates inflammatory response in rats with osteoarthritis	2017	184	30.67
2	Vasheghani et al	PPAR gamma deficiency results in severe, accelerated osteoarthritis associated with aberrant mTOR signaling in the articular cartilage	2015	145	18.13
3	Akasaki et al	FoxO transcription factors support oxidative stress resistance in human chondrocytes	2014	138	15.33
4	Wang et al	Leptin produced by joint white adipose tissue induces cartilage degradation via upregulation and activation of matrix metalloproteinases	2012	135	12.27
5	Chow et al	The role of inflammation in the pathogenesis of osteoarthritis	2020	128	42.27
6	Sun et al	The PI3K/AKT/mTOR signaling pathway in osteoarthritis: a narrative review	2020	116	38.67
7	Lim et al	Cytoprotective and anti-inflammatory effects of melatonin in hydrogen peroxide-stimulated CHON-001 human chondrocyte cell line and rabbit model of osteoarthritis via the SIRT1 pathway	2012	103	9.36
8	Liu et al	CTGF increases vascular endothelial growth factor-dependent angiogenesis in human synovial fibroblasts by increasing miR-210 expression	2014	99	11
9	Lee et al	Hypoxia enhances chondrogenesis and prevents terminal differentiation through PI3K/Akt/FoxO dependent anti-apoptotic effect	2013	87	8.7
10	Shen et al	Autophagy protects chondrocytes from glucocorticoids-induced apoptosis via ROS/Akt/FOXO3 signaling	2015	86	10.75

PI3K/AKT = phosphatidylinositol 3-kinase/protein kinase B, ROS = reactive oxygen species.

### 3.3. Publication output analysis

**Figure 2.**
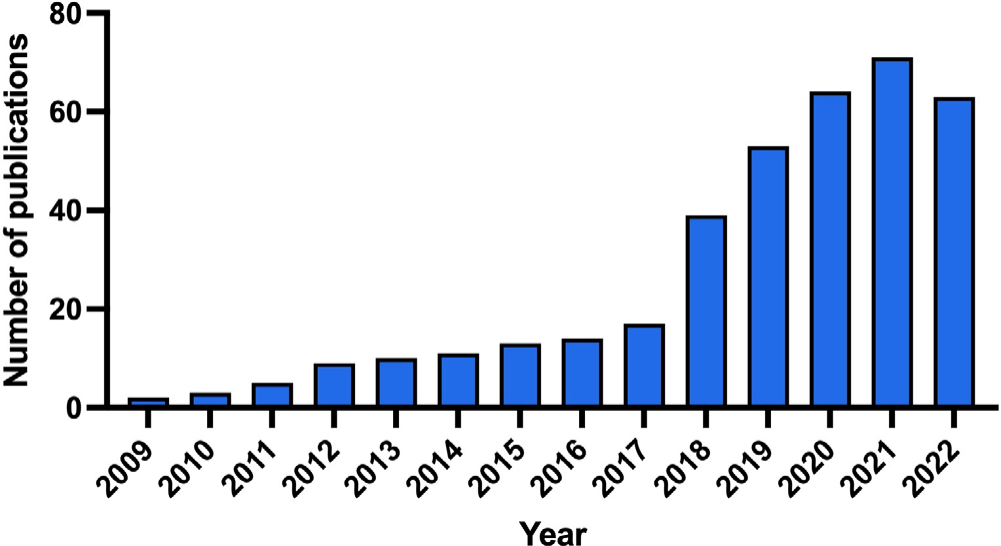
The publication year of the enrolled studies.

As shown in Figure 2, 374 articles on the PI3K/Akt signaling pathway in OA were published between 2009 and 2022. Before 2018, fewer than 20 articles were published annually. Since 2018, there has been an explosive and sustained increase in the number of published papers that peaked in 2021. These results indicated that the PI3K/Akt signaling pathway in OA has attracted increasing attention over the past decade and will remain under focus for a long time in the future.

### 3.4. Topic analysis

**Figure 3.**
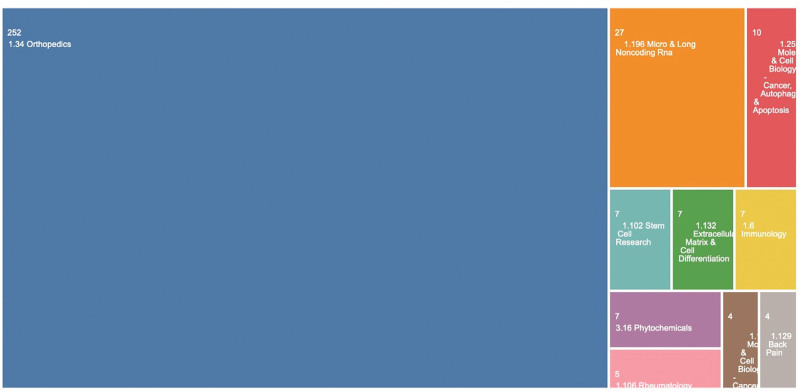
The topic of the article in selected references.

In all included articles, “orthopedics” was the dominant topic (252 of 374 [67.38%]), followed by “micro & long noncoding RNA” (27 of 374 [7.22%]), “mole & cell biology-cancer, autophagy & apoptosis” (10 of 374 [2.67%]), “stem cell research” (7 of 374 [1.87%]), and “extracellular matrix & cell differentiation” (7 of 374 [1.87%]) (Fig. 3).

### 3.5. Publication country/region and institutional analysis

**Figure 4.**
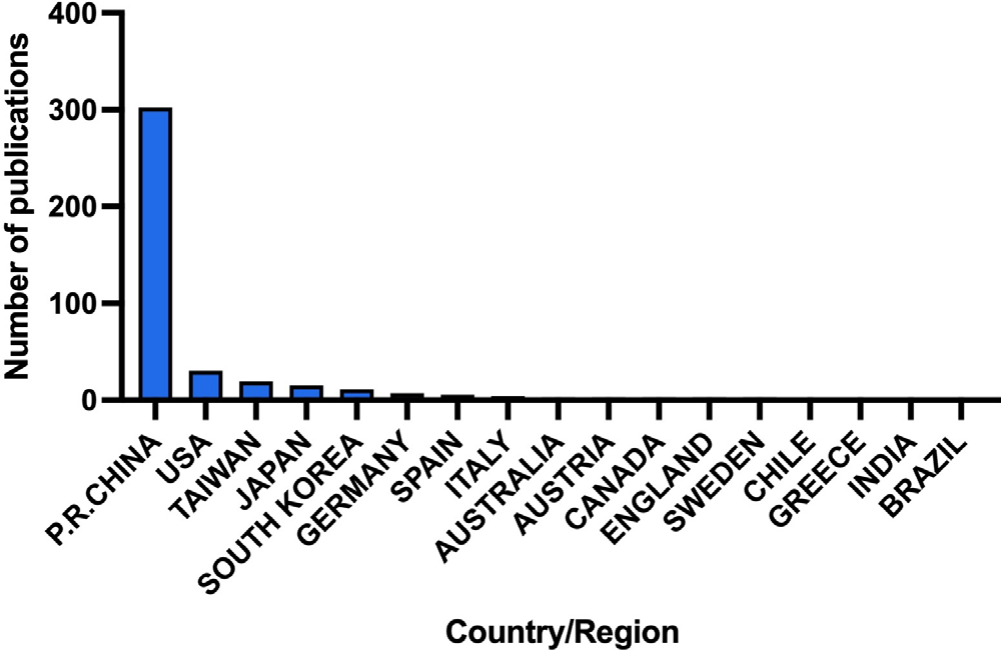
Publication countries/regions of enrolled studies.

The People’s Republic of China published the most articles (n = 302 [80.75%]), followed by the USA (n = 30 [8.02%]), Taiwan District (n = 19 [5.08%]), Japan (n = 15 [4.01%]), and South Korea (n = 11 [2.94%]). The top 3 countries published >95% of all relevant articles. Among the top 5 countries, 4 were from Asia (Fig. 4).

**Figure 5.**
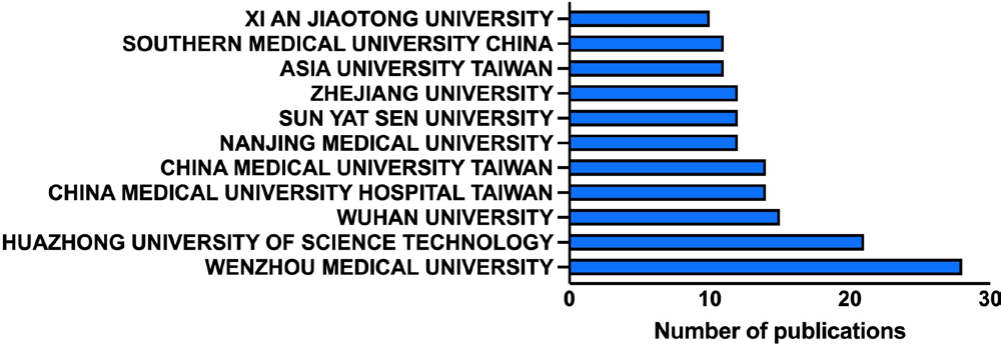
Publication institutions of enrolled studies.

Globally, 475 institutions independently or cooperatively published articles. Among them, 11 institutions that published 10 or more articles are listed (Fig. 5). The top 3 institutions were Wenzhou Medical University (n = 28 [7.49%]), Huazhong University of Science and Technology (n = 21 [5.62%]), and Wuhan University (n = 15 [4.01%]).

### 3.6. Publication journal analysis

**Figure 6.**
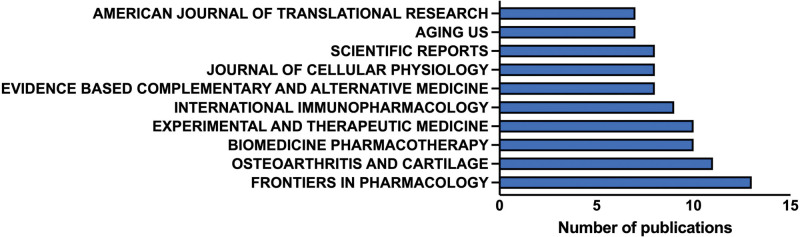
Publication journals of enrolled studies.

The enrolled 374 articles were published in 166 journals; the top 10 published journals are shown in Figure 6. Thirteen articles were published in *Frontiers in Pharmacology*, followed by *Osteoarthritis and Cartilage* (n = 11 [2.94%]), *Biomedicine Pharmacotherapy* (n = 10 [2.67%]), *Experimental and Therapeutic Medicine* (n = 10 [2.67%]), and *International Immunopharmacology* (n = 9 [2.41%]). Their IFs ranged from 2.650 (*Evidence-Based Complementary and Alternative Medicine*) to 7.507 (*Osteoarthritis and Cartilage*), with a median value of 5.835.

### 3.7. Bibliometric maps of co-citations

**Figure 7.**
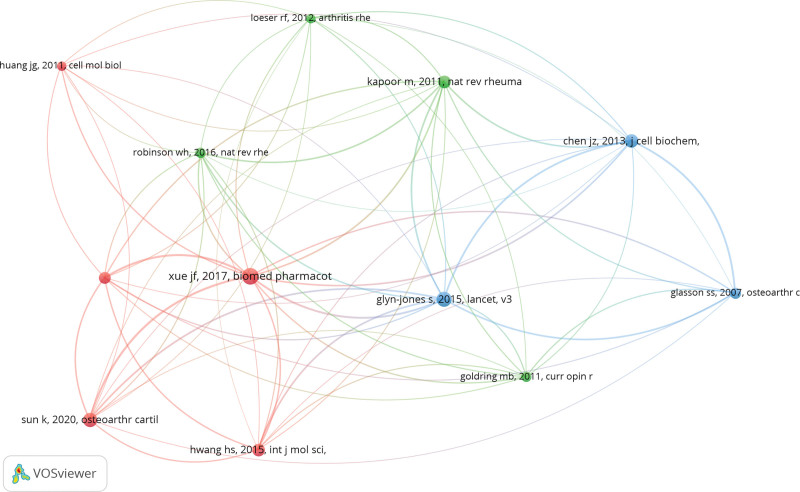
Mapping on co-cited references of studies.

The main principle of literature co-citation analysis is to infer the similarities between the studies based on the number of co-citations. The minimum number of citations of a cited reference was set as 20 of the 13,965 cited references, and 12 met the threshold and were subjected to the analysis. VOSviewer software was used to analyze and visualize the total strength of the co-citation links with each other’s cited reference. The minimum cluster size was set at 3 items. The first cluster included 5 publications that mainly focused on the effect of promoting or inhibiting the PI3K/Akt signaling pathway on OA progression. The second cluster included 4 publications that focused on the role of proinflammatory cytokines in the pathophysiology of OA. The third cluster included 3 publications and focused on establishing an OA research model (Fig. 7).

**Figure 8.**
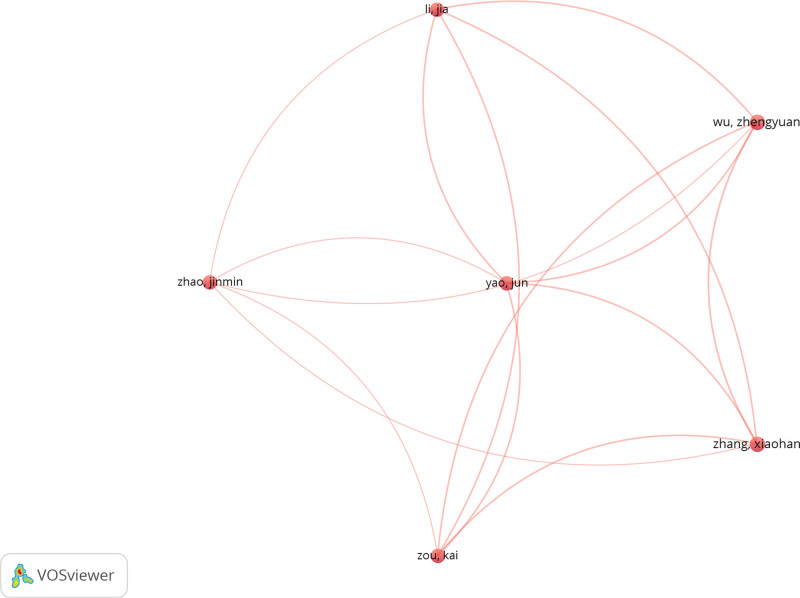
Mapping on co-cited authors of publications.

The authors’ co-citations were also analyzed. The minimum number of citations of each author was set at 5. Of the 2284 authors, 21 met the threshold and were included in the analysis. The total strength of the co-citation links with other authors was calculated for each of the 21 authors. Among all selected authors, Tang Chih-Hsin had the highest number of citations (n = 354). Both Zhengyuan Wu and Xiaohan Zhang had the highest total link strength of 23 (Fig. 8).

### 3.8. Bibliometric maps of co-occurrences

**Figure 9.**
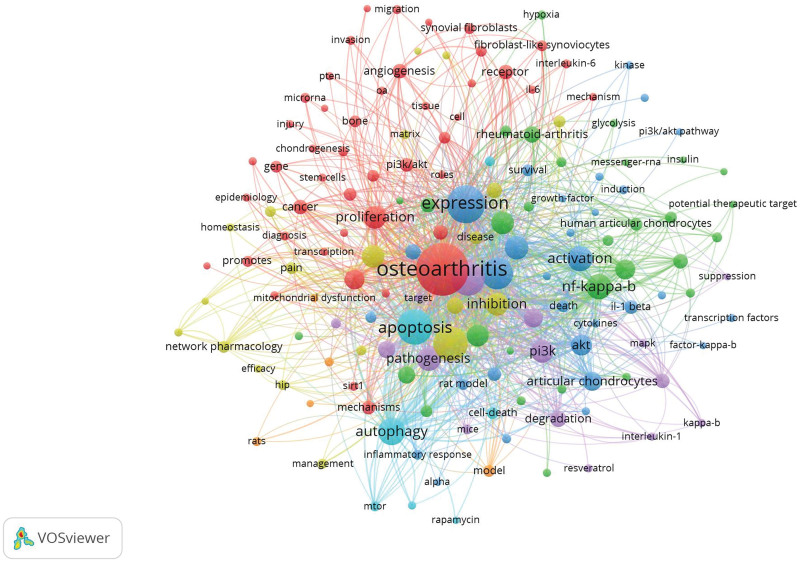
Mapping on the co-occurrence of keywords related to PI3K/Akt signaling on OA. OA = osteoarthritis, PI3K/Akt = phosphatidylinositol 3-kinase/protein kinase B.

**Figure 10.**
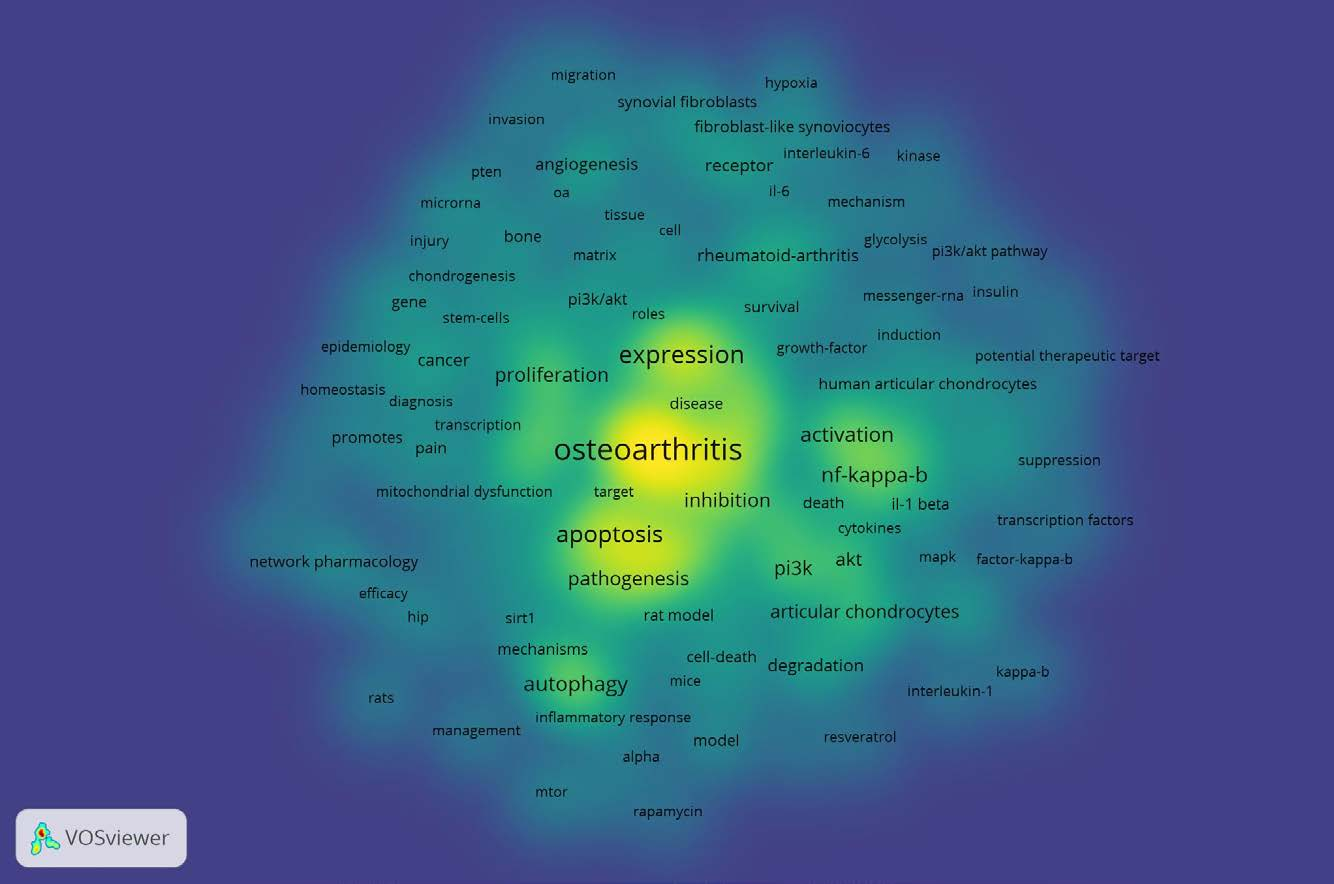
Visualization of time when a keyword appeared. Keywords in blue appeared later than that in yellow.

The co-occurrence of keywords related to the PI3K/Akt signaling pathway in OA was sent to VOSviewer software for analysis and visualization. The minimum number of occurrences of a keyword was set to 5 of the 1624 keywords; of them, 153 met the threshold. The top 5 keywords were “osteoarthritis,” “expression,” “apoptosis,” “chondrocytes,” and “inflammation,” with 252, 129, 116, 106, and 100 occurrences, respectively. The keywords “autophagy,” “mitochondrial dysfunction,” “inflammatory response,” “cartilage degeneration,” and “network pharmacology” appeared in the last few years, especially after 2020. All selected keywords were classified into 7 clusters. Cluster 1 consisted of 46 keywords that were mainly related to OA mechanisms. Cluster 2 consisted of 30 keywords that mainly discussed the OA microenvironment. Cluster 3 consisted of 30 keywords that mainly focused on the effect of OA progression by activating the PI3K/Akt signaling pathway. Cluster 4 consisted of 20 keywords that were mainly related to the influence of joint structure and microenvironment by targeting the PI3K/Akt signaling pathway. Cluster 5 consisted of 15 keywords that mainly discussed interpretation methods targeting the PI3K/Akt signaling pathway in the OA model. Cluster 6 consisted of 7 keywords that focused on the effect on cell proliferation and migration in the OA model by activating or inhibiting the PI3K/Akt signaling pathway. Cluster 7 consisted of 5 keywords that investigated the primary OA model establishment technique (Figs. 9 and 10).

## 4. Discussion

With the further study of OA, researchers have paid much more attention to the pathogenesis mechanism of such diseases, especially the molecular mechanisms. The PI3K/Akt signaling pathway plays a key role in regulating cell proliferation, growth, and apoptosis in the skeletal system. Hence, there has been a growing research trend toward decelerating OA progression via the PI3K/Akt signaling pathway. To the best of our knowledge, this is the first bibliometric study of the PI3K/Akt signaling pathway in OA. Numerous significant findings were obtained from this study.

### 4.1. Bibliometric findings

There was a growing trend in publication output, but the majority of the top 10 most-cited articles were published from 2013 to 2017. This could be explained by a common bias in bibliometric analysis, which is that the latest published articles did not have sufficient time to be cited. Therefore, the citation density of each article was calculated to assess its importance. Only 2 review articles published in 2020 had a higher citation density of 42.27 and 38.67. This may be because these 2 reviews focused on the effect of inflammation in the OA microenvironment and the role of the PI3K/Akt signaling pathway in OA. Other studies that investigated the treatment of the OA-targeted PI3K/Akt signaling pathway should consult these 2 reviews.

The People’s Republic of China ranked first in terms of the number of publications, while the USA ranked second. The PI3K/Akt signaling pathway was first identified by Professor Cantley from Weill Cornell Medical College in the USA, and its function in cancer progression was first introduced.^[14]^ This indicates that the People’s Republic of China dominates studies on the PI3K/Akt signaling pathway in OA. Considering that the population of Asia area ranked first in the world, and with the development of an aging population. The incidence of OA in Asia increased and is at a relatively high level around the world.^[15]^ Therefore, scholars in Asia are paying more attention to this disease caused by aging compared with other regions. Regarding publication institution analysis, the top 3 institutions were from the People’s Republic of China. This outcome is consistent with that of the publication country/region analysis. *Frontiers in Pharmacology, Osteoarthritis and Cartilage, Biomedicine Pharmacotherapy, Experimental and Therapeutic Medicine*, and *International Immunopharmacology* were the top 5 productive journals on the PI3K/Akt signaling pathway in OA, suggesting that more research on this topic will be published in these journals in the future. Although *Frontiers in Pharmacology* did not have the highest IF, due to its focus on the molecular mechanism of OA intervention, it had the highest number of publications. *Osteoarthritis and Cartilage*, the official journal of the Osteoarthritis Research Society International, fosters the cross-fertilization of findings from the clinical and basic sciences of the various disciplines involved. Researchers interested in the PI3K/Akt signaling pathway in OA should pay more attention to this journal.

### 4.2. Research focuses on PI3K/Akt in OA

Yoke Yue Chow and Kok-Yong Chin published a review entitled “The role of inflammation in the pathogenesis of osteoarthritis” in 2020, which had the largest citation density.^[16]^ In this review, they discussed the role of cytokines, immune cells, chemokines, matrix metalloproteinases (MMPs), signaling pathways, and miRNAs in OA. They concluded that inflammation plays an important role in OA pathogenesis. Several molecules released by chondrocytes, synoviocytes, and infiltrating immune cells are involved in regulating joint anabolism and catabolism processes. Their expression is, in turn, governed by a series of signaling pathways, including PI3K/Akt. “Inhibition of the PI3K/AKT/mTOR signaling pathway promotes autophagy of articular chondrocytes and attenuates inflammatory response in rats with osteoarthritis,” published in 2017 by Xue et al, was the most frequently cited article in the last decade.^[17]^ The authors indicated that inflammation could inhibit the proliferation and cell cycle of chondrocytes and reduce the autophagy rate. Moreover, inhibition of the PI3K/AKT/mTOR signaling pathway could promote autophagy of articular chondrocytes and attenuate the inflammatory response in the OA model.

In the OA progression microenvironment, the affected synovial cells and chondrocytes overproduce inflammatory mediators, such as interleukin (IL)-1β.^[18]^ However, IL-1β can rapidly stimulate PI3K and Akt phosphorylation, which activates the PI3K/Akt signaling pathway and triggers strong inflammatory responses.^[19,20]^ NF-κB, a main regulator of OA-related inflammatory mediators, is activated mainly by IκB kinase-mediated IκBa degradation and p65/RelA phosphorylation. Active PI3K and Akt are involved in NF-κB p65 phosphorylation and nuclear translocation, thereby promoting the secretion of inflammatory mediators.^[21]^ Besides, other cytokines, such as IL-1β, IL-6, and tumor necrosis factor (TNF)-α, diffuse into the cartilage, further stimulating the secretion of damage mediators in chondrocytes to amplify synovial inflammation and cartilage destruction.^[22]^ PI3Kδ and PI3Kγ are highly expressed in rheumatoid arthritis synovium and cultured synoviocytes and are closely related to the modulation of synovial inflammation.^[23]^ PI3Kδ is known to control the proliferation and migration of fibroblast-like synoviocytes (FLS), which may contribute to cartilage damage. Also, PI3Kδ expression is stimulated by inflammatory cytokines IL-1β and TNF-α. Targeting PI3Kδ could decrease inflammatory levels in synoviocytes.^[24]^ In addition, PI3Kγ deficiency reduces TNF-α-induced MMP expression and activation of AKT and extracellular regulated kinase (ERK) in synovial fibroblasts.^[25]^ IPI-145, a novel inhibitor of PI3Kδ and γ, represses inflammatory arthritis and cartilage damage.^[26]^ The PI3K downstream effector mTOR also serves as a proinflammatory response regulator in the synovium. TNF-α stimulates mTOR activation in cultured FLS, while mTOR negatively modulates TNF-upregulated expression of multiple proinflammatory cytokines or chemokines such as IL-6, IL-8, CCL20, CXCL11, MMP1, MMP-3, and prostaglandin-endoperoxide synthase 2 by limiting activation of NF-κB signaling to shift synovial FLS inflammation.^[27]^ In OA, the mTOR inhibitor rapamycin alleviates synovitis and IL-1β levels in synovial tissue in OA mouse knees.^[28]^ Taken together, these results suggest inhibition of PI3K/AKT could alleviate synovial inflammation in OA.

As the only cells in the cartilage, chondrocytes play an important role in cartilage and joint function. Reduced proliferation and excessive apoptosis of chondrocytes can cause cartilage degradation and accelerate OA progression.^[29,30]^ The PI3K/Akt signaling pathway is a vital regulator of chondrocyte proliferation and apoptosis. A study conducted on bakuchiol demonstrated its significant bioactivity towards chondrocyte proliferation via the PI3K-Akt and ERK1/2 pathways mediated by estrogen receptor activation and exhibited enhanced promotion of the remodeling of injured cartilage.^[31]^ Researchers have indicated that under severe hypoxic conditions, pretreatment with the mechano-growth factor E peptide upregulates chondrocyte proliferation through the PI3K-Akt and MEK-ERK1/2 signaling pathways.^[32]^ 17β-estradiol (E2) loss may be accompanied by an increased incidence of knee and hip OA.^[33]^ By contrast, E2-mediated PI3K/AKT activation significantly promotes cell proliferation in rat OA model chondrocytes.^[34]^ Administration of E2 also elevates the proliferation and viability of ATDC5 chondrocytes via this signaling.^[35]^ In addition, a study by Huang et al suggested that PTEN silencing increased AKT phosphorylation and promoted the proliferation of OA chondrocytes.^[36]^ Rapid cellular proliferation is also found during cartilage repair under exosomal CD73-mediated adenosine activation of AKT and ERK signaling while inhibitors of AKT or ERK phosphorylation repress exosome-mediated increases in cell proliferation.

In addition to regulating chondrocyte proliferation, PI3K/Akt regulates cartilage degradation by reducing apoptosis. Consistent with the inflammatory response, IL-1β can also induce chondrocyte apoptosis by suppressing PI3K and Akt.^[37]^ Thus, reduced IL-1β and other inflammatory mediator production or the active PI3K/Akt signaling pathway could be the methods of attenuating apoptosis. Han et al found that combined treatment with curcumin and probucol could protect chondrocytes from inflammatory cytokine stress by inhibiting the autophagy-related PI3K/Akt/mTOR pathway in vitro and in vivo, which might be a potential pharmaceutical value for OA prevention and therapy.^[38]^ A previous study reported that calycosin could inhibit IL-1β-induced degradation of the cartilage matrix, including the downregulation of MMP-3, MMP-13, collagen II, and aggrecan. Calycosin also inhibited NF-κB and PI3K/AKT in OA chondrocytes. Furthermore, calycosin inhibits IL-1β-induced chondrocyte apoptosis in a mouse OA model.^[39]^ Overproduction of NO in articular chondrocytes also induces apoptosis by modulating multiple intracellular signaling processes including PI3K/AKT signaling.^[40]^ Chun-Do et al reported that NO production in chondrocytes led to cell apoptosis, with downregulation of PI3K and AKT activities, while IGF-1 treatment blocked the process through PI3K and AKT activation.^[41]^ Not only that, but many bioactive compounds also including berberine^[37]^ and tormentic acid,^[42]^ exert similar anti-apoptotic effects via PI3K/AKT activation. Consistent with these results, other evidence reveals that activated PI3K/AKT can block OA chondrocyte apoptosis induced by TNF-α and lipopolysaccharides.^[43]^ Taken together, the PI3K/AKT signaling negatively modulates chondrocyte apoptosis under multiple pathological conditions and the activated signaling can protect against OA by reducing chondrocyte apoptosis.

Various studies on OA focused on reducing the inflammatory response, promoting cell proliferation, and attenuating apoptosis. As a major signaling pathway, PI3K/Akt regulates inflammatory mediator production, chondrocyte proliferation, and apoptosis. Improved cartilage function and delayed OA progression can be achieved by PI3K/Akt signaling pathway activation.

### 4.3. Emerging trends in PI3K/Akt in OA

Autophagy engulfs cytoplasmic proteins or organelles into vesicles, fuses with lysosomes to form autophagolysosomal, and degrades the contents therein to meet the metabolic needs of the cells themselves and renew some organelles. Failure to perform autophagy leads to elevated production of reactive oxygen species and mitochondrial dysfunction and can result in death at the cellular level.^[44]^ The PI3K/AKT signaling pathway promotes chondrocyte proliferation and inhibits autophagy. AKT activation can regulate chondrocyte survival, autophagy, and apoptosis through the phosphorylation of a variety of sub-phase target components, such as Bad, NF-κB, mTOR, and caspase-3, which mediate the process of cartilage tissue injury.^[45,46]^ Inhibition of the PI3K/AKT signaling pathway hinders proteoglycan synthesis and reduces chondrocyte survival rates.^[47]^ Moreover, inhibition of AKT activation is an important mechanism by which IL-β induces autophagy in chondrocytes, and AKT inhibitors can significantly block the effects of certain drugs on chondrocyte autophagy.^[48]^

Autophagy activity is reportedly inhibited by mTOR, which contains 2 heterogeneous complex compounds, mTORC1, and mTORC2. The negative regulating function of mTORC1 in autophagy has been demonstrated. The promotion of autophagy by AMPK is also indirectly achieved by inhibiting mTORC1 at the tuberous sclerosis complex 2 and Raptor levels. For example, in response to glucose starvation, the upstream pathway of mTORC1 activates AMPK and induces tuberous sclerosis complex 2 expression to inhibit mTORC1 activity. Moreover, mTORC1 indirectly loses its control over the autophagy inhibition sites of the downstream ULK autophagy initiation complex, thereby upregulating the autophagy activity of fine cells. In addition, mTORC2 is associated with cytoskeleton reassembly and cytoskeleton survival, and its phosphorylation activates AKT, thereby inhibiting autophagy.^[49]^ PI3K/AKT and mTOR can be combined to form the PI3K/AKT/mTOR signaling pathway and be involved in the pathways of various organisms to regulate cell proliferation, differentiation, and metabolism.

Network pharmacology, a novel research direction with broad prospects, is a new discipline based on the theory of systems biology that analyses the network of biological systems and selects specific signal nodes for the design of multitarget drug molecules. Network pharmacology emphasizes multi-pathway regulation to improve the therapeutic effect of drugs, reduce their toxic side effects, improve the success rate of new drug clinical trials, and reduce the cost of drug research and development. OA involves a variety of pathogenic factors, including environmental, genetic, and immune. OA development and progression may be related to a variety of targets or pathways, and a single component of traditional chemical drugs can only be effective on one or several targets and cannot achieve the goal of radical treatment. The active ingredients that inhibit OA can be explored and the mechanism of action explained through network pharmacology research, which has a guiding effect on clinical drug research. For example, a traditional Chinese medicine, baicalein, was recently identified, and its importance in the apoptotic signaling pathway in the treatment of OA was verified by combining network pharmacology and experimental validation.^[50]^ Similarly, a network pharmacological analysis of *Eucommia ulmoides*-*Radix Achyranthis Bidentatae* in the treatment of OA identified 50 active ingredients, including quercetin, kaempferol, wogonin, and baicalein, with important biological effects, and they were enriched in a series of signaling pathways including PI3K/AKT.^[51]^ Network pharmacology is a cross-discipline product. Based on network pharmacology, more knowledge of drug analysis, bioinformatics, biochemistry, and other disciplines will be combined. Network pharmacology can play a significant role in the pathogenesis of OA, treatment of the mechanism of anti-OA drugs, and development of new drugs.

Besides, we also searched relevant studies published after October 31, 2022. One study investigated the correlation of long non-coding RNA HOX transcript antisense RNA (lncRNA HOTAIR) with the PI3K/AKT pathway and clinical-related indicators in OA. Conclusively, lncRNA HOTAIR was highly expressed in OA-chondrocytes, which facilitated OA inflammatory responses by orchestrating inflammatory cytokines and the PTEN/PI3K/AKT pathway.^[52]^ Meng et al^[53]^ studied the connections of markers to chondrocyte autophagy and apoptosis in OA were also comprehensively explored in vitro using molecular biology approaches. PDK1 was finally identified as a diagnostic marker for OA. Inhibition of its expression can rescue OA-dysregulated autophagy and inhibit apoptosis by reducing the phosphorylation of PI3K/AKT signaling pathways. Researchers also found new cartilage-targeting drug delivery systems that are aimed at preventing reactive oxygen species production and angiogenesis may be of clinical significance for OA treatment. Curcumin suppressed angiogenesis and cartilage degradation partially via the reactive oxygen species-mediated PI3K-Akt signaling pathway.^[54]^ Consequently, the topic of PI3K/AKT on OA continued to be studied in the future.

## 5. Limitations

Although our bibliometric research yielded some valuable information, it involves some limitations: it included only English-language original articles, which may have led to overlooking high-quality literature in other languages in the field of the PI3K/Akt signaling pathway in OA and resulted in biased results; recently published studies included here did not have sufficient time to be cited, which may have relatively low values of the H-index and the total number of citations due to their short publication time, resulting in differences between the research results and the actual situation; and only the WOS database was used for the visualized analysis, which may lead to a biased picture. It would be better to use PubMed, Scopus, Embase, and other databases; otherwise, many important articles may be excluded.

## 6. Conclusions

In conclusion, our bibliometric study showed a growing trend in published articles related to the PI3K/Akt signaling pathway in OA. Our work provides a comprehensive list of publications on PI3K/Akt signaling in OA and recognizes the contributions of authors, countries/regions, and institutions. Furthermore, we summarized the emerging trends in the PI3K/Akt signaling pathway in OA. Inflammatory response, cartilage degeneration, and apoptosis remain central topics in OA. Research on autophagy, mitochondrial dysfunction, and network pharmacology is on the rise. The results obtained by a bibliometric and visual analysis must be supported by a large amount of comprehensive literature data. Given the increasing morbidity and burden of OA, the focus on PI3K/Akt pathway will continue to grow.

## Acknowledgments

The authors thank Miss Fan Ou for consulting relative image design and beautification.

## Author contributions

**Conceptualization:** Rui Zhang.

**Data curation:** Rui Zhang, Xiaoqin Deng.

**Formal analysis:** Xiaoqin Deng, Quan Liu, Xinxin Bai.

**Investigation:** Quan Liu, Xintian Zhang.

**Methodology:** Quan Liu, Xintian Zhang.

**Project administration:** Shaohuang Weng, Min Chen.

**Software:** Xintian Zhang, Xinxin Bai.

**Validation:** Xinxin Bai.

**Visualization:** Xinxin Bai.

**Writing – original draft:** Rui Zhang.

**Writing – review & editing:** Shaohuang Weng, Min Chen.

## Supplementary Material


